# Novel Principle of Contactless Gauge Block Calibration

**DOI:** 10.3390/s120303350

**Published:** 2012-03-08

**Authors:** Zdeněk Buchta, Šimon Řeřucha, Břetislav Mikel, Martin Čížek, Josef Lazar, Ondřej Číp

**Affiliations:** Institute of Scientific Instruments, Academy of Sciences of the Czech Republic, Královopolská 147, 612 64 Brno, Czech Republic; E-Mails: res@isibrno.cz (Š.Ř.); mikel@isibrno.cz (B.M.); cizek@isibrno.cz (M.Č.); joe@isibrno.cz (J.L.); ocip@isibrno.cz (O.Č.)

**Keywords:** low-coherence interferometry, gauge block, nanometrology

## Abstract

In this paper, a novel principle of contactless gauge block calibration is presented. The principle of contactless gauge block calibration combines low-coherence interferometry and laser interferometry. An experimental setup combines Dowell interferometer and Michelson interferometer to ensure a gauge block length determination with direct traceability to the primary length standard. By monitoring both gauge block sides with a digital camera gauge block 3D surface measurements are possible too. The principle presented is protected by the Czech national patent No. 302948.

## 1. Introduction

The use of length measuring instruments is a usual part of everyday life and these instruments are used in almost all branches of human activity. In general, a suitable length measuring instrument could be arbitrarily chosen with respect to the measurement range and accuracy needed. The accuracy of a length measuring instrument is ensured and verified by an instrument calibration process.

As for calibration of the length measuring instruments, there is no liberty in choice of a calibration instrument. Due to the basic principle of length metrology, the calibration instrument has to be more accurate, in ideal case by one order more than the instrument which is being calibrated.

In mechanical engineering practice, gauge blocks are at the end of the calibration chain. Gauge blocks are used as mechanical length etalons for an accurate calibration of length measuring instruments such as micrometers and calipers. Gauge blocks are made of steel, metal carbide or ceramics. In all cases, the gauge blocks eventually wear away for many reasons, including the mechanical contact between the gauge block and the calibrated instrument, material and form changes *etc*. For this reason, a regular gauge block calibration is necessary and it is a hot topic in fundamental and industrial metrology [1]. The methodology of gauge block calibration is defined by the international standard EN ISO 3650 describing two principal ways of calibrating gauge blocks.

The first method employs laser interferometry, white-light interferometry or multiwavelength interferometry [2,3]. The gauge block is adhered to the reference surface. Then the length of the gauge block is measured by one of the interferometric techniques mentioned above.

The second one is the contact method. In this case, the gauge block length is compared against a reference gauge block. The measured and reference gauge blocks are probed from both sides with a couple of incremental length gauges.

From a practical point of view, a contactless calibration technique could be a useful alternative to the techniques used nowadays. The main advantage is a possible automation of the calibration process.

The presented optical setup for contactless gauge block measurement is based on double-ended interferometry combining a laser and a white-light source and it is presented as an alternative to similar configurations published before [4–8]. The key advantage of the presented optical setup is its contactless measurement of the absolute gauge block length done as a single-step measurement giving complete information of the gauge block length, without any additional gauge block manipulation or any length comparison with a reference dimension. The measurement is fully automatic, with no operator influence.

Low-coherence interferometry is a powerful diagnostic technique which has become an attractive and a useful method for fast and accurate 3D inspection of macroscopic objects. Typical fields of application of white-light interferometry are contactless surface measurement of macroscopic objects, surface roughness and quality assurance, measurement of the thickness of thin films and optical tomography.

The principle of white-light interferometry stems from classical laser interferometry. The main difference between these two techniques is the type of the radiation source used. In case of white-light interferometry, a low-coherent light emitter (halogen lamp, xenon lamp, superluminescent LED diode) is used instead of a highly-coherent laser.

The principle of white-light interferometry is given by the white-light source. Its coherence length is a few micrometers (typically from 3 μm to 5 μm) [9,10]. The white-light source coherence length defines the range of the reference mirror movement in which the reference beam and the measuring beam interfere. This property predetermines the white-light interferometer to be an indicator of the reference interferometer arm and the measuring interferometer arm balance.

## 2. Experimental Setup

**Figure 1.**
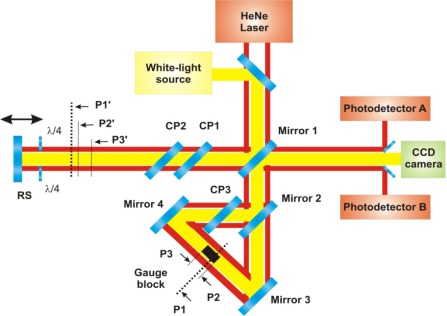
Optical setup for gauge blocks measurement. CP1, CP2 and CP3 are compensating plates, RS is a reference surface, λ/4 is a retardation quarterwave plate.

The presented optical setup combines a Michelson interferometer and a Dowell interferometer [11], placed in the reference arm of the Michelson interferometer. The principle of the measurement is illustrated in Figure 1. A parallel beam, generated by a white-light source, is divided into two parts by semireflecting mirror 1. The resulting measuring beam goes through a couple of compensating plates CP1 and CP2 and after that is reflected back by a reference surface RS.

The resulting reference beam of the Michelson interferometer plays the role of the primary beam for the Dowell interferometer. This beam is divided by mirror 2 into two beams going in the Dowell interferometer in opposite directions. One of them passes through compensating plate CP3 and is reflected by mirror 4 onto a gauge block face. The other beam passes through the beamsplitter (mirror 2) and then is reflected by mirror 3 onto the other face of the gauge block. One part of both mentioned beams illuminating the gauge block is reflected back by the gauge block faces and the other part of both beams pass alongside the gauge blocks.

At the output of the Michelson interferometer, there are five beams which could possibly interfere—the first one is the measuring beam of the Michelson interferometer reflected by the reference surface RS, then there is a pair of beams reflected by the gauge blocks and a pair of beams that pass alongside the gauge blocks.

The principle of the measurement is based on low-coherence interferometry taking advantage of low-coherence properties of the broadband light source. In the range of the movable reference surface shift, there are three positions where the white-light beams interfere. In the block diagram of the experimental setup shown in Figure 1, these positions are marked as P1′, P2′ and P3′.

In the P1′ position, the Michelson interferometer measuring beam interferes with the pair of beams passing alongside the measured gauge block. In fact, this is equivalent to a configuration with a mirror in a position marked as P1 (see Figure 1). P1 is at the mean optical path length of the ring interferometer, *i.e.*, the equidistance plane for both clockwise and anti-clockwise propagation after mirror 2, taking into account CP3. For the gauge block length measurement, it plays the role of the reference position.

As for positions P2′ and P3′, the Michelson interferometer measuring beam interferes with the beams reflected by the gauge block faces (marked as P2 and P3 in Figure 1). Then, the measured gauge block length is equal to the sum of distances between the measuring positions P2′ and P3′ of the reference position P1′.

**Figure 2.**
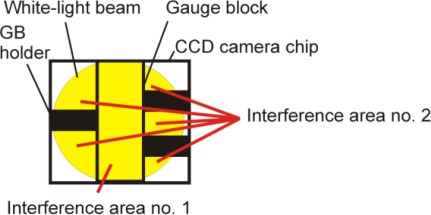
Sketch of the screenshot recorded by the CCD camera.

Figure 2 shows a sketch of a screenshot recorded by the CCD camera. There are two interference areas—the beams in the area no. 1 interfere in reference surface positions P2′ and P3′ and in the area no. 2, the beams interfere in reference surface position P1′. During a gauge block measurement procedure, only the interference signal from the relevant area of interference is recorded. The recorded data are used for complex gauge block analysis, namely gauge block length measurement, gauge block face surface measurement and for interference fringe shape analysis to evaluate the gauge block flatness distortion.

**Figure 3.**
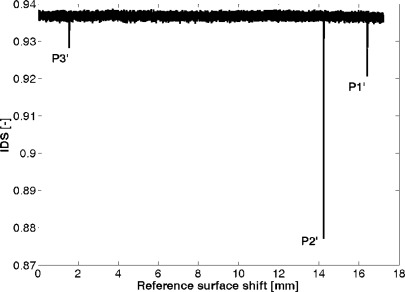
The Interference Detecting Signal (IDS) recorded during a pilot experiment with gauge block measurement. In the case shown in this figure, a gauge block with the length 17 mm was measured.

For illustration, an interference detecting signal (IDS) record is shown in Figure 3. The signal was recorded during a pilot gauge block measurement experiment and it represents a correlation coefficient value resulting from a comparison of two sequential samples of the output beam intensity. In the designed system, this signal is used for an automatic detection of the measuring positions. In the case shown in Figure 3, a gauge block with the length 17 mm was measured.

The gauge block measurement process is controlled by special software which was designed for the positioning control of the reference surface RS and the online acquisition of the signals from the photodetectors and a CCD camera. The measurement control process is based on monitoring the IDS signal during a fast positioning of the reference surface. The interference of the output beams is detected as a drop of the IDS signal. In this case, the motorized stage is switched off and the interference signal is measured by means of a PZT driven stage. The measuring positions P1′, P2′ and P3′ are localized by a central point detection algorithm implemented in the measurement control software. The measured values of positions P1′, P2′ and P3′ has to be corrected to respect the phase shifts caused by light beam reflections and parameters of the optical components used. The reference surface RS was made from the same material and by the same way like standard gauge block. Due to this fact, the estimated phase step on reflection is for positions P2′ and P3′ the same for the reference surface and for the gauge block and for steel gauge blocks it doesn’t need any other correction. In the case of position P1′, there is no additional reflection on the gauge block surface. This makes the measured P1′ position different from the right one and the total gauge block length seems to be longer than it in fact is. For P1′ correction, the missing phase step on reflection has to be taken into account—the phase step on reflection is 160° for the steel gauge block, which is the mean value calculated for the visible part of the optical spectrum using the optical constants published in [12]. Furthermore, the measured gauge block length (GBL) is calculated by the Equation (1):

(1) $$\mathrm{GBL}=\left|P1^{\prime }-P2^{\prime }\right|+\left|P1^{\prime }-P3^{\prime }\right|$$

By using of the CCD camera as a photodetector, this measuring principle allows measurement of the gauge block length in a number of points for verification of gauge block flatness and parallelism.

For precise gauge block measurements, it is necessary to acquire the measuring positions with nanometer range precision. In the designed experimental setup, this is ensured by an incremental laser interferometer which complements the low-coherence interferometer described above. As a source of laser radiation, a 633 nm He-Ne laser is used. Its output beam is collimated into an optical fiber, then split by a 50/50 fiber splitter and the resulting two laser parallel beams are used for the incremental interferometric measurement as shown in Figure 1. In general, it works as the classic Michelson interferometer. In the reference arm of the interferometer, the laser beams pass alongside the measured gauge block and at the output of the interferometer, they are combined with the laser beams passing through the measuring interferometer arm. The detection unit (DU) used as a photodetector A and B (see Figure 1) receives two associated beams from the measuring and reference arm of the interferometer [13].

**Figure 4.**
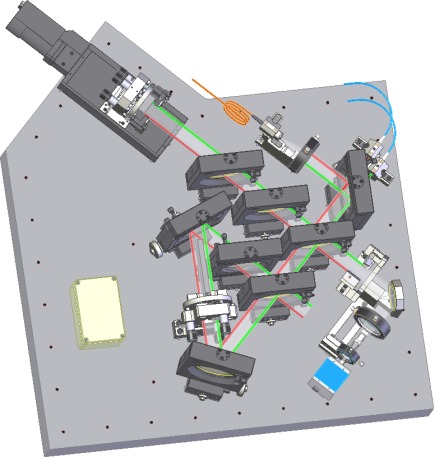
Sketch of the practical realization of the designed experimental setup.

The measurement setup is shown in Figure 4. It is arranged on a massive base plate made of gray iron with a thermal expansion coefficient of 12.3 × 10^−6^ K^−1^. The optical components are put into 3″ gimbal transmitting mounts TRANS 90G (OWIS) optimized for a maximum aperture. These transmitting mounts are combined with goniometers with a range of adjustment of ±10° designed and made for precise rotation of the optical components around a defined optical axis. The final adjustment can be fixed with a screw. The used transmitting mounts are made from aluminum alloy with a declared thermal expansion coefficient of 23.5 × 10^−6^ K^−1^ and the gauge block holder and the reference surface holder are made from steel with a thermal expansion coefficient of 10.6 × 10^−6^ K^−1^.

For the measurement process, the gauge block is placed by an automatic handling system into a gauge block holder supplemented by three PZT screw actuators (Thorlabs) used for gauge block adjustment before its measurement. The automatic handling system is designed for a set of 126 gauge blocks (0.5 mm to 100 mm) to allow the automatic contactless calibration of the complex gauge block set without a human operator.

The positioning of the reference surface is ensured by a combination of a LIMES 80-100-HiDS (OWIS) motorized positioning stage and a reference surface holder supplemented—like the gauge block holder—by three PZT screw actuators (Thorlabs). The travel of the Limes stage is 105 mm with a spindle pitch of 1 mm and 2,000 steps per revolution. The screw actuator with PZT provides 4 mm of manual coarse travel via a 0.25 mm pitch leadscrew. The 150 V internal piezo stack provides 15 μm of open-loop piezo travel and an 18-bit DA converter used in control electronics allows extremely fine positioning of the reference surface.

The combination of these two types of actuator meets the requirement for reference surface positioning in the 100 mm range with subnanometer resolution. In addition, using three PZT screws allows an online correction of the main stage pitch and yaw deviations.

Except for the main part of the optical setup, the measurement system was designed as a modular device. The sources of white-light and laser radiation and the detection system are made as separate modules to allow precise adjustment out of the system.

The base plate with the optical setup, shown in Figure 4, is oriented to the vertical position to respect the international standard EN ISO 3650 which requires the vertical calibrating position for gauge blocks of up to 100 mm in length.

The whole optical setup is covered by a double shielded housing for minimization of refractive index of air fluctuations. The value of the refractive index of air is calculated from measured atmospheric parameters by means of the Edlen formula and it is used for an online correction of the HeNe laser wavelength with 1 × 10^−7^ precision [14,15]. In addition to an ambient temperature measurement, there are three temperature sensors monitoring the base plate temperature for corrections of the optical path dilatation during the gauge block measurement process.

The described method was designed to be an alternative for the methods presently used for gauge block calibration. In comparison with the current methods, the key advantage of the presented optical setup is its contactless measurement of the absolute gauge block length, which is fully automatic, with no operator influence. Because of the automatic measurement process, a set of gauge blocks could be measured one by one without delay caused by waiting for temperature stabilization of each block. As for the time required for the diagnosis of one gauge block, it depends mainly on the set motorized stage and PZT scanning speed and this has to be set with respect to the electronics used (CCD cam frame rate, laser interferometer detection unit sensitivity and maximal sampling rate,…) and detection techniques (in our case, coherent correlation technique [16] for white-light fringe center detection and homodyne detection technique for laser interferometers). In the experimental setup in our lab, the diagnostics of a gauge block of 20 mm length takes about 120 s. During the pilot experiments, the repeatability of the measurement was tested. Generally, it depends on the measurement conditions, setup mechanical stability and also on used central fringe detection technique. For the described experimental setup, a standard deviation of six consecutive P1′ position measurements was 7.3 nm.

From the metrological point of view, the method’s relative uncertainty is a crucial factor. The most important factors making up the method’s relative uncertainty are the thermal expansion coefficients of the materials used (gauge block 10.6 × 10^−6^ K^−1^, base plate 12.3 × 10^−6^ K^−1^, gauge block holder and reference surface holder 10.6 × 10^−6^ K^−1^ and transmitting mounts 23.5 × 10^−6^ K^−1^) and the method of measurement of the refractive index of air.

The uncertainty of the refractive index of air is calculated as the combined standard uncertainty by the values presented in Table 1. The U(x_i_) is a standard uncertainty of an atmospheric parameter measurement, c_i_ is a relative change of an refractive index of air caused by change of an atmospheric value and U_i_(n) is a standard uncertainty of an atmospheric parameter.

The measurement uncertainty given by mechanical parameters of the experimental setup is calculated as a combined standard uncertainty using the values presented in Table 2.

In our setup version, the estimated relative uncertainty value is 2.0 × 10^−7^.

## 3. Conclusions

This paper presents a novel principle for contactless gauge block measurement using a combination of low-coherence interferometry and laser interferometry. The experimental setup combines a Dowell interferometer and a Michelson interferometer to ensure a gauge block length determination with direct traceability to the primary length standard. This setup was designed for contactless complex gauge block analysis providing information about gauge block length, gauge block faces surface profile (e.g., indication of scratches) and by analysis of the the interference fringes shape, also about the gauge block edge flatness distortion. The designed setup is supplemented by an automatic handling system designed for a set of 126 gauge blocks (0.5 mm to 100 mm) to allow the automatic contactless calibration of the complex gauge block set without a human operator. The described method was recently protected under Czech national patent No. 302948.

## Figures and Tables

**Table 1.** Refractive index of air—standard uncertainty.

	x_i_	U(x_i_)	*c_i_* = ∂*l*/∂*x_i_*	*U_i_*(*l*) / rel.
Atmospheric pressure	99 kPa	10	3E-9 /Pa	2.7E-08
Air temperature	20°C	0,01	1E-6 /K	1.0E-08
Relative humidity	60%	1	1E-8 /%	1.0E-08
CO_2_ concentration	700 ppm	10	1.5E-10/ppm	1.5E-09
Edlen formula	1.0002650	2.E-08	1	2.0E-08
				
RIA relative uncertainty				3.6E-08

**Table 2.** Uncertainty part given by mechanical parameters of the experimental setup

	x_i_	U(x_i_)	*c_i_* = ∂*l*/∂*x_i_*	*U_i_*(*l*)/ rel.
Gauge block thermal expansion	20°C	0.01	10.6E-6	1.1E-07
Air temperature	20°C	0,01	12.3E-6	1.2E-07
Mechanical components thermal expansion (gauge block holder, reference surface holder)	20°C	0,01	10.6E-6	1.1E-07
Total mechanical relative uncertainty				1.9E-07
